# Gastroenteropancreatic Neuroendocrine Neoplasms and Celiac Disease: Rare or Neglected Association?

**DOI:** 10.3390/jcm14030780

**Published:** 2025-01-25

**Authors:** Luca Pes, Anna La Salvia, Giovanni Mario Pes, Maria Pina Dore, Giuseppe Fanciulli

**Affiliations:** 1 Endocrine Clinic (Policlinico Sassarese), 07100 Sassari, Italy; 2 National Center for Drug Research and Evaluation, National Institute of Health (Istituto Superiore di Sanità), 00161 Rome, Italy; anna.lasalvia@iss.it; 3 Department of Medicine, Surgery and Pharmacy, University of Sassari, 07100 Sassari, Italy; gmpes@uniss.it (G.M.P.); mpdore@uniss.it (M.P.D.); 4 Department of Medicine, GI Section, Baylor College of Medicine, Houston, TX 77030, USA; 5 Neuroendocrine Tumor Unit, Department of Medicine, Surgery and Pharmacy, University of Sassari—Endocrine Unit, Azienda Ospedaliero-Universitaria of Sassari, 07100 Sassari, Italy; gfanciu@uniss.it

**Keywords:** celiac disease, neuroendocrine neoplasm, neuroendocrine tumor, gastroenteropancreatic neuroendocrine neoplasm, carcinoid

## Abstract

**Background:** Gastroenteropancreatic neuroendocrine neoplasms (GEP-NENs) are rare tumors originating from neuroendocrine cells in the gastroenteropancreatic system. They are increasingly recognized as being potentially associated with chronic intestinal inflammatory conditions, namely Crohn’s disease and ulcerative colitis. Celiac disease (CD) is an immune-mediated chronic gastrointestinal inflammation triggered by gluten in genetically predisposed individuals. This study aimed to explore the relationship between GEP-NENs and CD, providing a detailed review of the existing literature and addressing the (possible) gaps in current knowledge. **Methods:** We conducted an extensive search of international databases using relevant keywords, with the last update on 1 November 2024. A total of 19 studies, published between 1983 and 2024, were included: two prospective studies, five retrospective studies, and 12 case reports. **Results:** Overall, we included 107 GEP-NENs in our analysis. Among the 94 GEP-NENs identified in prospective and retrospective studies, the small intestine was the most common site (88.3%). The small intestine was also the most frequently reported site in the case report series (46.2%), accounting for 13 GEP-NENs in 12 patients with CD. **Conclusions:** Although most studies on the association between CD and GEP-NENs are heterogeneous, and while some lack crucial data, emerging evidence suggests that screening GEP-NEN patients for CD could offer valuable insights. Testing for the presence of CD might reveal whether the observed association is more than coincidental and possibly pave the way for exploring and understanding the role of chronic inflammation in the tumorigenesis of GEP-NENs in CD.

## 1. Introduction

Gastroenteropancreatic neuroendocrine neoplasms (GEP-NENs) are a group of rare tumors arising from neuroendocrine cells in the gastroenteropancreatic system. They are most frequently located in the small intestine, rectum, and pancreas, and they demonstrate a broad biological spectrum, ranging from indolent to highly aggressive tumors. Recent data suggest a rise in the global incidence, estimated at approximately 3.56 per 100,000/year, likely attributable to advancements in diagnostic technologies [1].

The WHO 2022 classification of GEP-NENs categorizes them into three groups based on the differentiation, mitotic rate, and Ki-67 index: well-differentiated neuroendocrine tumors (low-grade or G1 NET), well-differentiated neuroendocrine tumors (intermediate-grade or G2 NET), and poorly differentiated neuroendocrine carcinomas (high-grade or NEC). Additionally, the WHO 2022 classification introduces a new category for neuroendocrine tumors with an increased proliferation index, referred to as G3 NET [2].

GEP-NENs are increasingly recognized for their potential association with chronic inflammatory conditions, with a growing body of evidence suggesting that inflammation plays a key role in their tumorigenesis [3,4]. Chronic inflammation is thought to create a microenvironment conducive to genetic mutations and abnormal cellular processes, which can promote tumor development.

In the study by Vitale et al. [3], the complex relationship between inflammation and GEP-NENs was examined, noting that inflammatory cytokines like TNF-α and interleukins can activate the signaling pathways that lead to tumor growth, proliferation, and immune evasion. These pathways not only support tumor progression but also suppress anti-tumor immunity, creating an environment that favors the expansion of transformed neuroendocrine cells.

Similarly, Cigrovski Berkovic et al. [4] suggested that chronic inflammation and cytokines play a role in tumor development by modifying the immune landscape and promoting tumor survival. The inflammatory response can also facilitate tumor invasion and metastasis by altering the extracellular matrix.

Taken together, these findings highlight that inflammation may actively contribute to the initiation, promotion, and progression of GEP-NENs.

Several studies have explored the associations between conditions characterized by chronic inflammation of the gastrointestinal tract (Crohn’s disease and ulcerative colitis) and GEP-NENs, indicating a possible link between these conditions [5,6].

Celiac disease (CD) is a chronic immune-mediated enteropathy induced by gluten ingestion in genetically susceptible individuals (HLA DQ2 or DQ8 positive). The disease is characterized by chronic inflammation of the intestinal mucosa, driven by an abnormal immune response [7]. It is one of the most common life-long disorders worldwide, with a prevalence ranging between 0.7% and 2.9% in the general population [8].

Despite the documented links between GEP-NENs and chronic inflammatory conditions, no comprehensive reviews currently analyze, compare, or summarize the potential relationship between GEP-NENs and CD.

## 2. Aim of the Study

In our narrative review, we aimed to investigate the potential link between these two conditions by analyzing the available evidence in detail and addressing potential gaps in the current understanding of their interrelationship.

## 3. Materials and Methods

We performed an extensive search for studies in the international databases PubMed, Web of Science, and Scopus using the following keywords: celiac disease, celiac sprue, neuroendocrine tumor, neuroendocrine carcinoma, carcinoid tumor, gastroenteropancreatic neuroendocrine tumor, and gastroenteropancreatic neuroendocrine neoplasm. We only included articles published in English. The search was last updated on 1 November 2024.

Between 1980 and 2022, various editions of the WHO classification were published, containing differences in the nomenclature and tumor grading [9]. The terminology used in the published studies has been maintained as such in the review and is indicated in quotation marks.

## 4. Results

### 4.1. Overview of the Studies 

**Figure 1:**
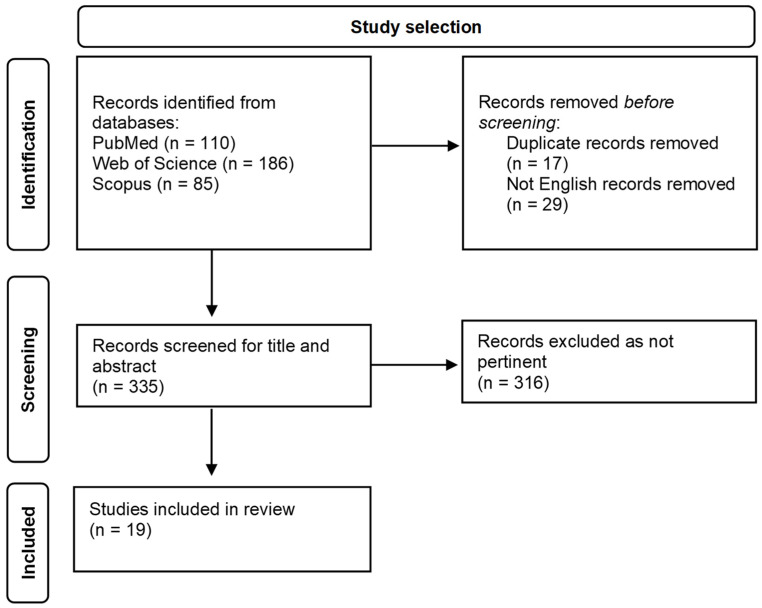
Study selection flowchart.

We identified 19 relevant studies published between 1983 and 2024, comprising two prospective studies, five retrospective studies, and 12 case reports. The study selection flowchart is reported in Figure 1.

The results are summarized in Table 1 (prospective and retrospective studies) and Table 2 (case reports).

#### 4.1.1. Prospective and Retrospective Studies

Swinson et al. [10] analyzed the clinical records of 235 patients with “both CD and histologically confirmed malignancies across various sites”. Among the 259 neoplasms in this cohort, only one gastric “carcinoid” was reported. No further details about the tumor were provided in the study.

In the study by Askling et al. [11], based on the records of 11,019 CD patients from a national register, the presence of a single GEP-NEN, identified as “mixed carcinoid-adenocarcinoma” of the small intestine was indicated. This study did not provide additional information about the tumor.

The design of Howdle and coworkers’ study [12] markedly differed from the other studies reviewed. Rather than focusing on a population of patients already diagnosed with CD, this study investigated 288 patients with small intestinal malignancies. The research was performed by sending a card to UK clinicians to report newly diagnosed cases of primary small bowel malignancy. Following notification of a case, a second form was sent to the reporting clinician requesting an anonymous identifier, the type of small bowel malignancy, and whether there was a diagnosis of CD. Strikingly, 124 out of 288 patients (43.1%) were found to have CD. The number of “carcinoids” detected among these CD patients was notably high, with 68 GEP-NENs identified in 124 CD patients (54.8%). The involved sites were as follows: duodenal (n = 2), jejunal (n = 9), ileal (n = 54), and carcinoids with an unknown primary site (n = 3). Interestingly, no GEP-NENs were found in the non-CD patients.

Tomba et al. [13] studied a case series of 53 patients classified as patients with “CD with alarm symptoms/signs or non-responsive disease”. This group included patients diagnosed after age 50, with persistent or recurrent gastrointestinal symptoms despite adherence to a gluten-free diet, or non-compliance with the diet, or the presence of alarm symptoms or signs. Investigations (i.e., colonoscopy, small bowel capsule endoscopy, CT, MRI) were carried out when clinically indicated. Within this population, three tumors were identified, including one case of a “well-differentiated neuroendocrine tumor” of the ileum (1.9%).

Perez-Cuadrado-Robles and coworkers [14] employed inclusion criteria similar to those used by Tomba et al. [13]. They examined a case series of 189 CD patients who underwent small bowel capsule endoscopy due to “alarm symptoms” or “refractory disease”. Among these patients, one “neuroendocrine tumor” of the ileum was reported, yielding a prevalence of 0.5%. No further details regarding the neoplasm were reported.

In the study by Emilsson et al. [15], using data from a national database, 48,119 CD patients were evaluated, and three small intestine “carcinoids” were identified, representing a prevalence of 0.006% within the population under study.

In the latest reported study, conducted by Haider and colleagues [16] utilizing data from the National Inpatient Sample [17], the authors assessed the prevalence of malignant neoplasms in 108,052 patients with CD compared to a control cohort of non-CD patients, matched for age, sex, and race. The study identified 14 cases of “malignant carcinoid tumors of the small intestine” and 5 cases of “malignant carcinoid tumors of the large intestine”, accounting for 0.01% and 0.005% of the CD cohort, respectively. No significant differences in the incidence of these tumors were found between the CD patient and control populations. In addition, the study reported, in the table describing the prevalence of malignant neoplasms (CD vs. controls), a statistically significant difference in the incidence of “other neuroendocrine tumors”, with 74 cases in the CD population compared to 50 in the control group (*p* = 0.03). It is important to note that, since the study did not specify the tumor sites, these tumors cannot be classified with reasonable certainty as GEP-NENs.

**Table 2:** Characteristics of the selected case reports on the association between celiac disease and gastroenteropancreatic neuroendocrine neoplasms.

First Author	Year	Diagnosis of CD	Diagnosis of GEP-NEN	First Diagnosis (CD vs. GEP-NEN)	Active /Inactive CD at the Diagnosis of GEP-NEN *	Number of Tumors in Patients with CD (Non-GEP-NENs and GEP-NENs)	Number of GEP-NENs	Gender	Age (Diagnosis Of GEP-NEN)	Site of GEP-NEN	Definition of GEP-NEN	Size of GEP-NEN (cm)	Grade and/or Differentiation Details	Ki-67 or Mitosis	Metastases	Metastases Site
Hallert C [18]	1983	Histological	Histological	CD	Active	1	1	M	69	Ileum	Carcinoid (malignant)	NR	NR	NR	Yes	Omental tissues
Gardiner GW [19]	1985	Histological	Histological	GEP-NEN	NR	1	1	M	57	Small intestine (Jejunum-Ileum)	Carcinoid	9.0	Poorly differentiated (atypical)	Frequent mitosis	No	NA
Levi S [20]	1988	Histological	Histological	CD	Inactive	1	1	F	49	Pancreas	Somatostatinoma (malignant)	3.0	NR	NR	No	Liver
Frick EJ Jr [21]	2000	Histological	Histological	CD	NR	1	1	F	43	Ampulla of Vater	Somatostatinoma (neuroendocrine tumor)	2.4 × 1.7 × 1.7	NR	Rare mitosis	No	NA
Sottile R [22]	2001	Histological	Histological	GEP-NEN	NR	1	1	M	65	Ileum	Neuroendocrine tumor	9.0 × 5.0 × 3.0	NR	NR	Yes	Liver, omental tissues, intestine
Kimchi NA [23]	2005	Histological	Histological	Contextual	Active	3	2	M	74	Ileum	Carcinoid (2 tumors)	1.0–1.2	NR	Low mitotic activity	NR	NR
Hlivko J [24]	2008	Histological	Histological	CD	NR	1	1	M	84	Jejunum	Neuroendocrine carcinoma	4.0	High-grade poorly differentiated	NR	Yes	Lymph nodes
Manjunath S [25]	2009	Histological	Histological	CD	NR	1	1	F	74	Appendix, terminal ileum and caecum	Goblet cell carcinoid	NR	NR	NR	Yes	Lymph node
Gundling F [26]	2014	Histological	Histological	Contextual	Active	1	1	F	37	Pancreas	Neuroendocrine carcinoma	4.0 × 3.0 × 3.0	G2 non-functioning	Ki-67 12%	Yes	Lymph node
Kathpalia P [27]	2016	Histological	Histological	CD	NR	1	1	F	24	Rectum	Carcinoid tumor	0.3	G1	Ki-67 < 1%	No	NA
Çetin D [28]	2017	Histological	Histological	Contextual	Active	1	1	M	41	Rectum	Neuroendocrine tumor	1.0	G1	Ki-67 1–2%	No	NA
Dhillon H [29]	2021	Diagnosis described as “positive Abs + symptoms + subtle histological changes”	Cytological (EUS)	CD	NR	1	1	F	49	Pancreas	VIPoma	1.5	Well differentiated, low grade	NR	Yes	Liver

Abbreviations: Abs, antibodies; CD, celiac disease; EUS, endoscopic ultrasound; GEP-NEN, gastroenteropancreatic neuroendocrine neoplasm; NA, not applicable; NR, not reported; * diagnosis based on serological and/or histological criteria.

Among the GEP-NENs (n = 94) reported in the seven mentioned studies, the most frequent site was the small intestine (n = 83; 88.3%). Other sites included the large intestine (n = 5), unknown (n = 3), duodenum (n = 2), and stomach (n = 1). Such data, particularly the high proportion of small intestine neoplasms, must be interpreted with caution due to the studies’ selection criteria, especially those of Howdle [12], where neoplasms are, by definition, located in the small intestine (as per the inclusion criteria).

#### 4.1.2. Case Reports

Among these 12 studies [18,19,20,21,22,23,24,25,26,27,28,29], a total of 13 GEP-NENs were identified in twelve patients, with one patient exhibiting two different lesions [23].

The demographic data were as follows: six females and six males; age range 24–84 years (mean 56, median 53).

Among the GEP-NENs reported, the most frequent site was the small intestine (n = 6; 46.2%). Other sites included the pancreas (n = 3), rectum (n = 2), ampulla of Vater (n = 1), and “appendix, terminal ileum and caecum” (n = 1).

Interestingly, 3 of the 12 patients presented with hormonal syndrome, somatostatinoma in two cases and VIPoma in one. Unfortunately, the data regarding the tumor differentiation, grade and proliferation index are missing. Data on metastatic lesions were reported for 11 out of the 12 patients: 6 patients out of 11 had metastasis (54.6%). When reported, the metastases sites included the lymph nodes, liver, omental tissue, and intestine.

## 5. Discussion

The associations between GEP-NENs and chronic intestinal inflammatory conditions [5,6] might provide a theoretical framework for understanding the potential role of chronic inflammation in GEP-NEN development in the context of CD.

Chronic inflammation is widely recognized as a critical driver of tumorigenesis, creating a microenvironment that actively promotes the development and progression of cancer. This inflammatory environment is characterized by an array of pro-inflammatory cytokines, such as IL-1β, IL-6, and TNF-α, which play pivotal roles in driving cellular processes like proliferation, survival, and metastasis. Immune cells, particularly macrophages and neutrophils, are integral components of this process, contributing to both the initiation and the progression of tumors through the release of cytokines and growth factors that foster a tumor-supportive microenvironment. These immune cells also generate reactive oxygen species (ROS) and reactive nitrogen species (RNS) during the inflammatory response, which are responsible for DNA damage and the accumulation of oncogenic mutations, thereby enhancing genetic instability [30]. Moreover, inflammatory signaling pathways, such as NF-κB and STAT3, further amplify tumorigenesis by promoting tumor cell proliferation, survival, and immune evasion. These pathways facilitate immune suppression, allowing tumor cells to avoid immune surveillance and enhancing their ability to metastasize. The NF-κB pathway, for example, is particularly involved in regulating genes that support inflammation, while STAT3 is crucial for the survival and expansion of tumor cells, particularly under inflammatory conditions [31]. This chronic inflammatory state, through these pathways, creates a dynamic environment that favors tumor growth and progression. In the tumor microenvironment, inflammation-induced mediators are essential in shaping tumor development. Tumor-associated macrophages (TAMs) and myeloid-derived suppressor cells (MDSCs) are particularly influential, as they secrete pro-tumor cytokines that not only enhance angiogenesis, thereby supporting tumor vascularization, but also suppress anti-tumor immune responses. This dual function of TAMs and MDSCs results in a favorable niche for tumor cells, promoting their survival, growth, and capacity for metastasis. Additionally, the chronic inflammatory milieu further enhances the ability of tumor cells to evade immune detection, thus allowing for more aggressive tumor behavior [30,31].

Interestingly, several haplotypes have been identified as strongly associated with CD, with HLA-DQ2 and HLA-DQ8 being the most prevalent. Among these, HLA-DQ2 is considered the most strongly associated haplotype and plays a central role in the genetic predisposition to CD [7] In 2014, Landerholm and colleagues conducted a study that demonstrated a significant overrepresentation of HLA-DQ2 in patients with neuroendocrine tumors of the small intestine (SI-NETs) [32]. This finding suggests a potential genetic link between CD and the development of SI-NETs, highlighting the possibility that individuals with certain genetic markers, particularly HLA-DQ2, may have an increased risk of both conditions.

The two mechanisms—chronic inflammation and genetic susceptibility—are likely not independent but may interact synergistically, promoting tumorigenesis [33]. In genetically predisposed individuals, chronic inflammation may establish a permissive microenvironment that supports the clonal expansion of cells, thereby contributing to cancer development. In the context of SI-NETs in CD patients, however, the exact nature (if any) of the interaction between inflammation and genetic susceptibility has not yet been investigated.

As an additional point, elevated concentrations of 5-hydroxyindoleacetic acid (5-HIAA), the principal metabolite of serotonin, have been observed in individuals with CD [34], suggesting a dysregulation in the function of enterochromaffin cells (ECs), which are responsible for producing serotonin in the gastrointestinal tract. This finding points to a potential alteration in the enteroendocrine system in CD, which may contribute to the gastrointestinal symptoms commonly experienced by patients. Furthermore, gluten challenge studies have demonstrated an increase in the EC population in CD patients, particularly following gluten exposure [35]. This observation implies that gluten-induced inflammation may influence EC proliferation or activity, thereby linking the immune response to changes in EC dynamics. While the hypothesis that inflammation, EC dysregulation, and the possible neoplastic transformation of ECs are interconnected is compelling, a causal relationship has yet to be established.

Overall, our review included 107 GEP-NENs in patients with CD, providing a comprehensive review of the available literature about this challenging and largely unexplored issue.

Ninety-four GEP-NENs were detected among 167,791 patients across seven relevant prospective and retrospective studies.

The global incidence of GEP-NENs is currently estimated to be approximately 3.56 cases per 100,000 individuals annually [1]. In parallel, CD is recognized as one of the most prevalent chronic conditions worldwide, with prevalence estimates ranging from 0.7% to 2.9% of the general population [8]. As of 2024, the global population is estimated to be around 8.2 billion individuals. Given these statistics, assuming that GEP-NENs and CD are unrelated conditions, a straightforward calculation based on their incidence and prevalence rates, combined with the current global population, suggests that between 2050 and 8364 new cases of coexisting GEP-NENs and CD would be expected to occur annually. However, if there exists any form of interdependence between these two conditions—such as a shared underlying pathophysiological mechanism, genetic predisposition, or both—the actual number of new cases of coexisting GEP-NENs and CD is likely to be substantially higher than the calculated estimate. In fact, a potential association could indicate a considerably greater frequency of concurrent cases, which underscores the importance of exploring the possible interactions between these diseases.

However, in our view, it was not feasible to extract meaningful data on the prevalence of GEP-NENs in CD from these studies, given the heterogeneity of numerous variables included (see Table 1).

Only three studies [11,15,16] evaluated the presence of GEP-NENs in CD, without specifying additional clinical conditions. Two studies investigated a selected subgroup of CD patients with “alarm symptoms/signs or non-responsive disease” [13,14] and thus non-representative of the “standard” population of CD patients. One study explored patients with “CD and malignancy” of any site [10]. Additionally, one study did not examine a population of CD patients but rather a cohort with small intestine malignancy, subsequently evaluating whether these patients also had CD [12]. Thus, the variety in the studied population represents a significant limitation to establishing, even tentatively, a clear association between these two conditions.

Furthermore, the diagnostic methods used for the diagnosis of both CD (serological, histological) and GEP-NENs (biopsy, histology, clinical diagnosis), as well as the diagnostic sequencing (whether CD or GEP-NENs were diagnosed first), are inconsistent across the studies, having considerable implications for the interpretation of the results. These three factors are fully addressed in only two studies [13,15], while they are entirely absent in three studies [11,12,16], with variable combinations in the remaining studies.

The basic demographic features (e.g., gender, age at diagnosis) of patients with GEP-NENs were completely reported in only two of the seven studies [12,13]. Information on the tumor size, grade, differentiation, Ki-67 percentage, mitotic count, and metastasis (absence, presence, and location, if applicable) was incomplete in most studies.

The section on case reports describes 12 patients over a span of 38 years. The most striking finding appears to be the localization, with small intestine involvement in 46.2% of the cases. However, these case reports exhibit notable heterogeneity: specifically, in seven patients the first diagnosis was CD, in two patients the first diagnosis was a GEP-NEN, and in the remaining three patients the diagnosis was contextual.

Where available, considering both prospective/retrospective studies and case reports, data on the sex distribution of GEP-NEN patients with CD revealed a higher prevalence in males (n = 49) versus females (n = 35). According to updated data from a systematic review and meta-analysis, the prevalence of CD is higher in female vs. male individuals (0.6% vs. 0.4%; *p* < 0.001) [36]. Therefore, our observation of an increased the male sex in GEP-NENs with CD sound intriguing, but further confirmation in larger series is needed.

Interestingly, in both case series and case reports, the small intestine was the most frequent site of GEP-NENs, suggesting the potentially intriguing biological significance of this finding, which may deserve further dedicated studies.

The best approach to assessing the potential association between GEP-NENs and CD is a crucial issue.

Although epidemiological analysis using the ICD codes for GEP-NENs and CD may seem to be, and likely will be in the future, the best approach for investigating a potential association between these two conditions, there are significant concerns about the use of ICD codes in epidemiological studies, particularly for identifying GEP-NENs and other NENs. Currently, the ICD codes do not precisely identify GEP-NENs due to their broad classification, which can introduce errors and inconsistencies in data collection. For instance, the ICD-10 code C7A.8 denotes “Other malignant neuroendocrine tumors” but it includes a wide range of tumors without specifying their exact location or characteristics. Likewise, the code D3A.8 refers to “Other benign neuroendocrine tumors”, again lacking critical specificity. These broad classifications make it difficult to conduct accurate analyses of the relationship between GEP-NENs and CD. To enhance the reliability and accuracy of epidemiological studies, it is essential to refine the coding system, which could provide more detailed and useful data for future research [37,38].

One potential approach could be a systematic screening for GEP-NENs in patients with CD. However, this approach (e.g., CT scans, MRI, capsule endoscopy, and conventional endoscopy) would involve extensive, risky, and costly diagnostic procedures. Furthermore, it could lead to the detection of numerous incidental GEP-NENs, which would offer no clear benefit to the CD patient. Additionally, performing a similar study in a control population of non-CD individuals would be ethically untenable, making the results from the CD population highly uncertain.

Alternatively, would a systematic search for CD in patients with GEP-NENs be a more appropriate approach? This strategy might have a stronger basis, as suggested by the study of Howdle performed in patients with small intestinal malignancies [12], and might reveal a (possible) association between these two conditions. GEP-NENs are indeed rare, yet there may be a true association with CD, like the relationship between CD and both gut lymphoma and adenocarcinoma of the small intestine [7].

## 6. Conclusions

Although most studies on the association between CD and GEP-NENs are heterogeneous, and some crucial data are missing, emerging evidence suggests that screening GEP-NEN patients for CD could offer valuable insights. Testing for the presence of CD might reveal whether the observed association is more than coincidental and possibly pave the way for exploring and understanding the role of chronic inflammation in the tumorigenesis of GEP-NENs in CD.

## Figures and Tables

**Table 1:** Characteristics of the selected studies evaluating the association between celiac disease and gastroenteropancreatic neuroendocrine neoplasms.

First Author	Year	Design of Study	Population Under Investigation (Patients)	Diagnosis of CD	Diagnosis of GEP-NEN	First Diagnosis (CD vs. GEP-NEN)	Number of Patients Under Study	Number of Patients with CD	Number of Tumors in Patients with CD (Non-GEP-NENs and GEP-NENs)	Number of GEP-NENs	Gender	Age (Diagnosis Of GEP-NEN)	Site of GEP-NEN	Definition of GEP-NEN	Size of GEP-NEN (cm)	Grade and/or Differentiation Details	Ki-67 or Mitosis	Metastases	Metastases Site
Swinson CM [10]	1983	Retrospective	“CD and malignancy” (any site)	Histological	Histological	NR	235	235	259	1	NR	NR	Stomach	Carcinoid	NR	NR	NR	NR	NR
Asklink J [11]	2002	Retrospective	CD	NR	NR	NR	11,019	11,019	249	1	NR	NR	Small intestine	Mixed carcinoid– adenocarcinoma	NR	NR	NR	NR	NR
Howdle PD [12]	2003	Prospective	Small intestine malignancy	NR	NR	NR	288	124	124	68	M (n = 41) F (n = 27)	Mean 65, range 38–87	Duodenum (n = 2) Jejunum (n = 9) Ileum (n = 54) unknown (n = 3)	Carcinoid	NR	NR	NR	Yes (37/68)	NR
Tomba C [13]	2014	Prospective	“CD with alarm symptoms/signs or non-responsive disease”	Histological	Histological	CD	53	53	3	1	F	47	Ileum	Neuroendocrine tumor	1.0	Well differentiated	NR	No	NA
Perez-Cuadrado-Robles E [14]	2018	Retrospective	“CD with alarm symptoms/signs or non-responsive disease”	NR	Histological	CD	189	189	8	1	NR	NR	Ileum	Neuroendocrine tumor	NR	NR	NR	NR	NR
Emilsson L [15]	2020	Retrospective	CD	Histological	Histological	CD	48,119	48,119	80	3	M (n = 2) F (n = 1)	NR	Small intestine	Carcinoids	NR	NR	NR	NR	NR
Haider MB [16]	2024	Retrospective	CD	NR	NR	NR	108,052	108,052	15,884	19	NR	NR	Small intestine (n = 14) Large intestine (n = 5)	Malignant carcinoid	NR	NR	NR	NR	NR

Abbreviations: CD, celiac disease; GEP-NEN, gastroenteropancreatic neuroendocrine neoplasm; NR, not reported.

## Data Availability

Not applicable.
